# Impact of Biomass Drying Process on the Extraction Efficiency of C-Phycoerythrin

**DOI:** 10.3390/biotech12020030

**Published:** 2023-04-23

**Authors:** Ariadna H. Vergel-Suarez, Janet B. García-Martínez, Germán L. López-Barrera, Andrés F. Barajas-Solano, Antonio Zuorro

**Affiliations:** 1 Department of Environmental Sciences, Universidad Francisco de Paula Santander, Av. Gran Colombia No. 12E-96, Cúcuta 540003, Colombia; 2 Department of Chemical Engineering, Materials, and Environment, Sapienza University, Via Eudossiana 18, 00184 Roma, Italy

**Keywords:** cyanobacteria, phycobiliproteins, colorants, biomass dehydration

## Abstract

Drying the biomass produced is one of the critical steps to avoid cell degradation; however, its high energy cost is a significant technological barrier to improving this type of bioprocess’s technical and economic feasibility. This work explores the impact of the biomass drying method of a strain of *Potamosiphon* sp. on the extraction efficiency of a phycoerythrin-rich protein extract. To achieve the above, the effect of time (12–24 h), temperature (40–70 °C), and drying method (convection oven and dehydrator) were determined using an I-best design with a response surface. According to the statistical results, the factors that most influence the extraction and purity of phycoerythrin are temperature and moisture removal by dehydration. The latter demonstrates that gentle drying of the biomass allows removing the most significant amount of moisture from the biomass without affecting the concentration or quality of temperature-sensitive proteins.

## 1. Introduction

Phycoerythrin (PE) is one of the most exciting colorants that can be obtained from some genera of red algae and cyanobacteria. The red-purple protein (depending on the strain obtained) has demonstrated its antimicrobial and antioxidant capabilities [1], which makes it a new and interesting bioresource in the production of novel high-end products for nutraceutical, cosmetic, and pharmaceutical companies. According to the literature, three different forms of PE can be found, which strictly depend on the sourced organism. R-phycoerythrin (R-PE) can be found in Rhodophytes (red algae, both micro- and macroalgae), B-phycoerythrin (B-PE) can be found in strains of *Porphyridium* sp., and C-phycoerythrin (C-PE) can be found in cyanobacteria [2].

One critical step in the biotechnological application of any inner metabolite derived from microbial biomass is the destruction of the cell wall. The rupture of the cell wall, especially in microalgae and cyanobacteria, is a critical step in the downstream process for valorizing multiple metabolites synthesized by different strains [3]. In this case, due to the unique nature of the multi-layered cyanobacterial cell wall, the biomass must first be pre-treated to allow a fast and efficient extraction of all the different metabolites [4].

Over the years, several pre-treatment methods have been designed and tested for the efficient extraction of phycobiliproteins. According to the scientific literature, the most frequently reported pre-treatment methods are freeze–thaw [5,6,7,8], enzymatic treatment [7,9,10], and drying of biomass [3,11,12,13,14,15]. In the case of freeze–thaw, the biomass is first dried with a mild method such as freeze drying or spray drying; then, the biomass is rehydrated using a known volume of cold phosphate buffer or a mixture of green solvents and mixed using a bead-miller, sonicator, or another equipment available. After this process, the mixture is frozen (10–24 h) and thawed (several times) until the complete content of phycocyanin is extracted [6,7,8].

In the enzymatic treatment, the concentrated biomass (via centrifugation or another harvesting technique) is treated with a known concentration of an enzyme under specific conditions (biomass/enzyme ratio, pH, mixing, aeration, etc.). In this case, the cyanobacterial biomass does not require a drying process, reducing the energy necessary to extract the synthesized phycocyanin [9]. Finally, drying can be considered a pre-treatment since the process can modify the morphology of the cell wall, which facilitates the penetration of solvents for the extraction of the selected metabolites [3]. However, drying is, in fact, a time-consuming and energy-intensive process that allows the removal of the water concealed in the cytoplasm and the excess water (also known as moisture) trapped within the harvested biomass. Biomass moisture acts as a natural barrier against desiccation [4]. In most microalgae and cyanobacteria, the extracellular substances secreted by the cells absorb a known amount of water, which helps the cells to overcome drought season. In the case of the biotechnological usage of cyanobacterial biomass, the harvesting process allows the cells to trap a significant amount of water, which may become a barrier for the proper extraction of the target metabolites.

One of the main advantages of removing excess moisture is the reduction of the degrading time of the biomass and its metabolites by inner enzymes and exogenous organisms, which in turn increases its shell life [12], allowing the transportation and handling of the produced biomass until it reaches its final destination. There are some drawbacks that make this process less preferable. Some of the most high-valuable metabolites produced by microalgae and cyanobacteria (including PE) are thermolabile; therefore, temperatures above 50 °C can rapidly degrade their content and purity [16]. Additionally, extended operational times (more than 20 h) can eventually degrade other metabolites such as carbohydrates, lipids, and carotenoids. Consequently, low-intensive methods for the removal of excess moisture within cyanobacterial biomass are needed.

Highly advanced techniques, such as freeze and spray drying, are available at different scales, including lab and industrial scales. These two techniques are preferred over more heat-intensive alternatives such as a convection oven because they use low temperatures and longer times, which ensure the slow removal of moisture while maintaining the stability and quality of different metabolites, including C-PE [17,18,19,20]. However, these two techniques can be expensive on the industrial scale, which, in turn, will increase the cost of the final product to be obtained [21,22].

One possibility is the dehydration of biomass. Unlike an oven, a food-grade dehydrator can slowly remove the excess moisture concealed within the biomass using low temperatures (40–70 °C) and extended drying times. In a previous work, Barajas-Solano [3] proved for the first time that a food-grade dehydrator could effectively remove the excess moisture within the biomass on a thermotolerant strain of *Oscillatoria* sp., while improving the extractability of different phycobiliproteins, including phycocyanin, allophycocyanin, and phycoerythrin. However, the available information on the application of this technology on other cyanobacterial strains and the possible effect of this type of drying on the quality of their metabolites is scarce. Therefore, the objective of this study is to determine if the drying process (drying temperature, drying time, and equipment employed) can influence the concentration and purity of C-Phycoerythrin (C-PE) from a newly isolated strain of thermotolerant *Potamosiphon* sp.

## 2. Materials and Methods

### 2.1. Strain

*Potamosiphon* sp. UFPS003 was isolated from a thermal spring near the city of Cucuta (Colombia) and kept in solid slants of BG-11 media at the INNOValgae collection (UFPS, Cucuta, Colombia) (https://www.innovalg.com (accessed on 13 March 2023)). The strain was initially cultured in a 1 L Schott GL45 glass flask with 0.5 L of working volume of liquid BG-11 media [23]. The strain was kept mixing through the constant injection of filtered air with 1% (*v*/*v*) CO_2_ at a flow rate of 0.3 L min^−1^, a photoperiod of 12:12 h at 100 µmol m^−2^ s^−1^, and a temperature of 27 ± 1 °C, for 15 days.

### 2.2. Experimental Design

The effect of drying in the concentration and purity of C-PE was tested using a three-level, three-factor (two numeric, one categoric factor), I-optimal design (two central points, five blocks, and twenty-six runs) coupled with surface response in Design-Expert^®^ software (Version 22.0.2, Stat-Ease Inc., Minneapolis, MN, USA). Table 1 shows the levels studied for each factor, while Table 2 presents the resolved design.

### 2.3. Drying Equipments

For the experiment, 2 drying equipment, a 60 L 1.2 kW lab-grade oven with forced convection (FD56, Binder, Germany), and a 500 W Hamilton Beach^®^ 32,100 a food-grade dehydrator (Hamilton Beach^®^, Hamilton Beach Brands Holding Company, Glen Allen, VA, USA) was tested. On an external level, freshly harvested biomass without any kind of drying was also used, along with freshly harvested biomass.

### 2.4. Culture Conditions

For each experiment, *Potamosiphon* sp. UFPS003 was cultured (in triplicate) in 0.5 L flasks with a working volume of 0.2 L of BG-11 culture media. Each experiment was grown for 20 days, mixed through the injection of filtered air with 1% (*v*/*v*) CO_2_ at a flow rate of 0.15 L_air_ min^−1^, with a photoperiod of 12:12 h at 100 µmol m^−2^ s^−1^ 27 ± 1 °C.

### 2.5. Biomass Drying, C-PE Extraction, and Quantification

After 20 days of culture, each flask was removed from the air inlet and allowed to self-precipitate (20 min). The excess culture media (upper part of the flask) was gently removed by sterile pipetting in sterile hood. The concentrated biomass (approximately 100 mL at the bottom of the flask) was transferred to 500 mL centrifuge-resistant plastic flask and centrifuged at 3600 rpm (20 °C, 20 min).

The different samples obtained were poured into non-stick, food-grade silicone molds [3] and dried in either a 60 L 1.2 kW lab-grade oven with forced convection (FD56, Binder, Germany), and a 500 W Hamilton Beach^®^ 32,100 a food-grade dehydrator (Hamilton Beach^®^, Glen Allen, VA, USA) following the experiments of Table 2. The biomass obtained after drying was recorded using a digital balance and divided into seven equal samples. The dehydrated biomass and the fresh samples from the experimental design (Table 2) were subjected to extraction (one sample plus six replicates) using the method previously described by Barajas-Solano [3]. Briefly, an amount of dried biomass was mixed with a volume of cold phosphate-buffered solution (0.05 M, pH 6.8) until it reached a biomass/solvent ratio of 0.26% (*w*/*v*) and a known amount of glass beads (0.5 mm diameter) until reaching a glass beads/biomass ratio of 14.9% (*w*/*v*). The biomass/glass beads/buffer mixture was thoroughly mixed using an automatic vortex (Multi Reax, Heidolph, Germany) at 1486 rpm for 30 min. To allow better extraction of the C-PE, the biomass/glass beads/buffer mixture was stored in a refrigerator at 4 °C (24 h). Finally, the deep-purple extract rich in C-PE (upper part of the sample) was separated from the exhausted biomass (lower part of the sample) by centrifugation (3400 rpm, 30 min, 20 °C).

The deep-purple supernatant was collected and measured in a spectrophotometer at different wavelengths (620, 652, 562, and 280 nm). The concentration of C-PC, APC, and PE was calculated using Equations (1)–(3) described by Bennett and Bogorad [24]. The purity of C-PC, APC, and PE was determined using Equations (4)–(6) proposed by Patil [25] and Antello et al. [26]. The average of the results obtained for each experiment was used to perform the ANOVA analysis according to the Design-Expert^®^ software. (Version 22.0.2, Stat-Ease Inc. Minneapolis, MN, USA).

(1) $$\mathrm{PC}\;[g/L]=\frac{{OD}_{620}-0.474\left({OD}_{652}\right)}{5.34}$$

(2) $$APC\;[g/L]=\frac{{OD}_{652}-0.208\left({OD}_{620}\right)}{5.09}$$

(3) $$\mathrm{PE}\;[g/L]=\frac{{(OD}_{562}-2.41\left(P-PC\right)-0.849(APC))}{9.62}$$

(4) $$\mathrm{PC}\;[\mathrm{purity}]=\frac{{\mathrm{OD}}_{620}}{280}$$

(5) $$APC\;[\mathrm{purity}]=\frac{{\mathrm{OD}}_{652}}{280}$$

(6) $$\mathrm{PE}\;[\mathrm{purity}]=\frac{{OD}_{562}}{280}$$

### 2.6. Evaluation of Moisture Ratio and Drying Rate

The moisture ratio and drying rate curves were obtained using freshly produced samples of *Potamosiphon* sp. biomass. A total of 40 samples (1 original plus 39 replicates) of 400 mg of concentrated biomass were dehydrated according to the optimized conditions. The samples were removed every 30 min, and their weights were recorded to obtain the moisture ratio and drying rate. The moisture content of cyanobacterial biomass at time *t*, *X_t_* (g water × g dry matter^−1^) can be defined according to Equation (7).

(7) $$X_{t}=\frac{m_{t}-m_{d}}{m_{d}}$$

The moisture content can be expressed as dimensionless moisture ratio (MR) following Equation (8):

(8) $$M_{R}=\frac{X_{t}-X_{e}}{X_{i}-X_{e}}$$

where *X_i_* and *X_e_* are initial and equilibrium moisture contents, respectively. Considering longer drying times, *X_e_* is considered too small compared to *X_i_* and *X_t_*; therefore, Equation (5) can be simplified into Equation (9).

(9) $$M_{R}=\frac{X_{t}}{X_{i}}$$

The drying rate (*D_R_*) of algal biomass can be calculated from the moisture content of microalgae using Equation (10).

(10) $$D_{R}=\frac{X_{t+∆t}-X_{t}}{{∆}_{t}}$$

where *D_R_* is drying rate (g water/g dry matter × min), $X_{t+∆t}$ is the moisture content at time *t* + *dt* (g water × g dry matter^−1^), and dt is time increment (min) [27].

## 3. Results

### 3.1. Effect of Multiple Parameters on the Drying in the Extraction Quantity of C-PE

The results of the ANOVA analysis (Table 3) on the effect of multiple variables (Drying temperature (A), Drying time (B), and Drying method (C)) on the drying in the extraction quantity and purity of C-PE from *Potamosiphon* sp. shows that in the case of the concentration of C-PE, the model F-value of 225.98 implies the model is significant. There is only a 0.01% chance that an F-value this large could occur due to noise; therefore, the results did not happen by chance. On the other hand, the *p*-values obtained (<0.05) for the different variables analyzed show that the drying temperature (A) and the Drying method (C) used are statistically significant variables that affect the extraction of the colorant protein. The Lack-of-Fit F-value of 0.23 implies the Lack of Fit is insignificant relative to the pure error. The latter means there is a 98.30% chance that a Lack-of-Fit F-value this large could occur due to noise, which means that the model fits.

The result obtained for the predicted R² of 0.9577 is in reasonable agreement with the adjusted R² of 0.9772, with a difference of less than 0.2. Additionally, the Adeq Precision of 28.543 indicates an adequate signal-to-noise ratio.

### 3.2. Effect of Multiple Parameters on the Drying in the Purity of C-PE

The results of the ANOVA analysis on the effect of multiple variables (Drying temperature (A), Drying time (B), and Drying method (C)) on the drying in the extraction quantity and purity of C-PE from *Potamosiphon* sp. (Table 4) shows that in the case of the purity of C-PE, the model F-value of 24.11 implies the model is significant. The latter shows that there is only a 0.01% chance that an F-value this large could occur due to noise; therefore, the results did not happen by chance.

On the other hand, the *p*-values obtained (<0.05) for the different variables analyzed show that the drying temperature (A) and the Drying method (C) used are statistically significant variables that affect the extraction of the colorant protein. The Lack-of-Fit F-value of 0.488 implies the Lack of Fit is not substantial relative to the pure error. The latter means that there is a 48.81% chance that a Lack-of-Fit F-value this large could occur due to noise, which implies that the model obtained from the data has a good fit.

The result obtained for the predicted R² of 0.7675 is in reasonable agreement with the adjusted R² of 0.5783, with a difference of less than 0.2. Additionally, the Adeq Precision of 10.97 indicates an adequate signal-to-noise ratio.

**Figure 1:**
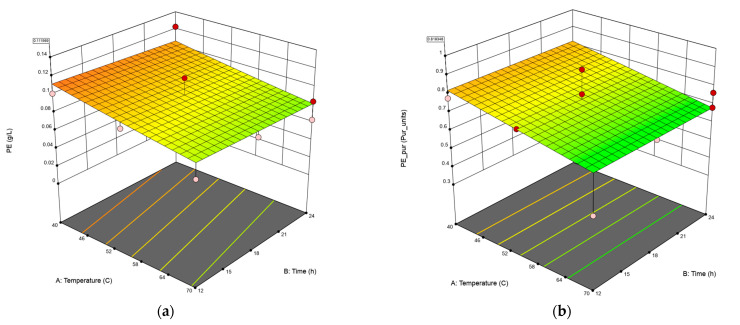
Surface response (three-dimensional plots) of the model equation fitted to the data on extraction (g/L) (**a**) and purity of C-PE (**b**).

The surface responses (three-dimensional plots) obtained from the extraction and purity of C-PE are shown in Figure 1a,b. According to the results, a low temperature (40 °C) and low times (12 h) of drying using a food-grade dehydrator maximizes both extraction and purity of C-PE.

Table 5 represents the best scenario that maximizes C-PE concentration and purity using the analyzed variables; in this case, the best drying method is the food-grade dehydrator. The obtained conditions were tested using fresh biomass obtained from 12 flasks (500 mL with 200 mL of working volume, grown at a photoperiod of 12:12 h, 100 µmol m^−2^ s^−1^ 27 ± 1 °C, for 20 days). The dried biomass was extracted using the method described in Section 2.5. The concentration and purity of C-PE were calculated according to Equations (3) and (6).

**Figure 2:**
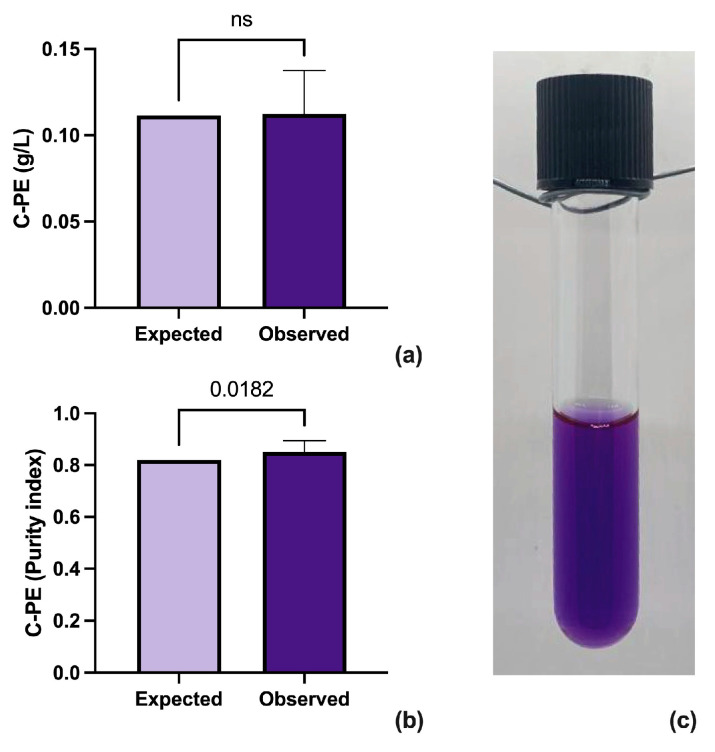
The unpaired *t*-test between the expected and observed results from extraction (**a**), purity (**b**) of C-PE, and unpurified sample of extracted C-PE from *Potamosiphon* sp. (**c**).

The results obtained after the drying of the biomass, according to Table 3, were analyzed using an unpaired *t*-test in the software PRISM (Version 9.5.1, GraphPad Software, Boston, MA, USA). (Figure 2a,b). The analysis shows that the proposed method can reduce the moisture of the biomass without affecting the concentration and purity of C-PE. Additionally, the brilliant purple color of the protein is maintained (Figure 2c).

### 3.3. Evaluation of Moisture Ratio and Drying Rate

**Figure 3:**
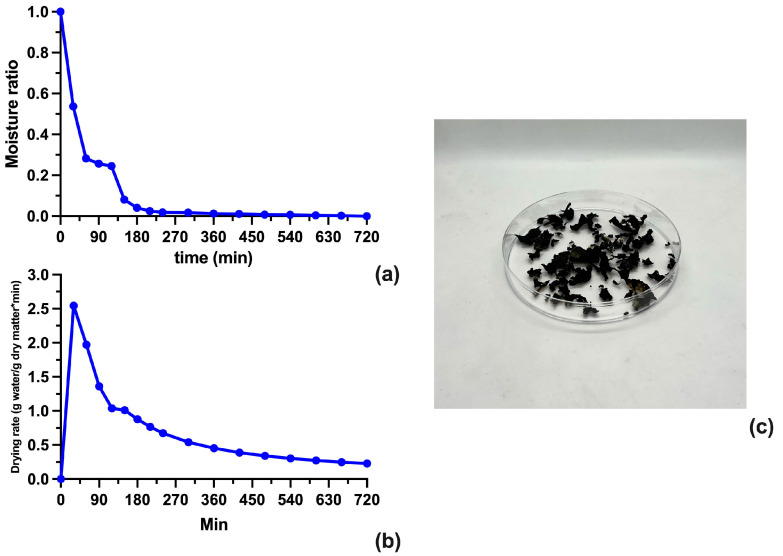
Moisture ratio curve (**a**), drying rate of cyanobacterial biomass curve (**b**), and dried biomass (**c**).

The moisture ratio, the drying rate curves, and the dried biomass are shown in Figure 3a–c, respectively. The moisture ratio curve shows the change in moisture content over time, indicating that the moisture drops fast within the first hour, followed by slower drying up to the fourth hour until reaches constant moisture since the eighth hour of drying. The drying rate curve, on the other hand, shows the rate at which moisture is being removed from the material over time; in this case, after 30 min of drying, the process was entirely in the falling rate period. This implies that the dehydration of the cyanobacterial biomass was controlled by the diffusion of moisture from the inner part of the biomass paste to the surface [27]. Finally, the dehydrated biomass possesses a flake-like texture which is an easy grind for the extraction of phycoerythrin.

## 4. Discussion

The primary source of industrial-grade PE are strains from the red algae *Porphyridium* sp., especially *P. cruentum* [18,28,29,30,31,32] and *P. purpureum* [17,18,19,33,34]. However, this genus is also known as a primary source of sulfated EPS; therefore, the available protocols for extracting PE are adapted to the unique requirements of these unicellular red algae, which makes the extraction and recovery process tedious and time-consuming [35]. Over the last years, the research on the isolation of novel strains, especially from cyanobacteria with the ability to over-produce C-PE, has obtained an interesting number of strains that may make them promising suitable C-PE producers. Strains from the genus *Tolypothrix* sp. *Nostoc* sp. *Anabaena* sp. and *Pseudanabaena* sp. have been previously reported to have higher C-PE content than some red algal strains [36,37]. On the other hand, the most studied cyanobacteria are the species belonging to the genus *Arthorspira* sp. (commercially known as *Spirulina*). The species of this genus are industrially known as the primary source of phycocyanin-C (rather than PE); therefore, the up-to-date available information is not properly focused on cyanobacterial producers of C-PE. *Potamosiphon* sp. is a newly described genus, which was isolated from samples obtained from a small coastal stream in subtropical north-eastern Australia in 2019 [38], but in there is no report on the ability of this genus to produce specific metabolites. To the author’s knowledge, so far, there have been no other reports on their presence outside the initial sampling site. Therefore, this is the first report on the biotechnological application of *Potamosiphon* sp. to produce C-PE.

Due to their peripheral position in the phycobilisomes [15] and their physicochemical characteristics, the phycobiliproteins are thermolabile metabolites [39]; therefore, most of the literature published in recent years focused mainly on the extraction and purification of phycobiliproteins rather than the effect of the dehydration of biomass on the concentration and purity of those specific proteins. Most of those studies avoid using harsh drying equipment such as a convection oven while preferring high-cost technologies such as freeze drying or spray drying [40,41,42,43], which will ensure that most of the initial content o phycobiliproteins are still present in the biomass.

Most of the literature cited so far focuses on the concentration and extractability of phycocyanin, especially C-PC, but, so far, to the best of the author’s knowledge, there is no available data on the role of drying in the quantity and purity of C-PE.

So far, the analysis and selection of novel and affordable technologies for removing excess moisture in cyanobacteria can only be found in a handful of scientific papers. According to Rezvani et al. [12], a continuous infrared-reflectance window™ dryer coupled with a double-pass photovoltaic-thermal solar collector can be an alternate solution for drying the *Spirulina* sp. biomass without degrading highly valuable compounds such as phycocyanin. Another example is the application of accelerated solar dryers [15], which can significantly reduce the moisture of *Spirulina* sp. However, the technologies proposed by Rezvani et al. [12] and Stramarkou et al. [15], which rely on the intensity of solar radiation available at the time, the latter can be a problem for year-round operation systems located in regions outside tropical areas. Additionally, another problem might be the excess heat in summer, which can significantly reduce the content of phycocyanin and other thermal-sensitive metabolites such as carotenoids, proteins, carbohydrates, secondary metabolites such as Indole-3-Acetic-Acid, polyhydroxybutyrates, and others that can be synthesized by the microorganism.

Another possibility is the usage of a cast-tape dryer [14]. Unlike a solar collector, a cast-tape dryer places the biomass in a band circulating in a high-temperature zone, which helps to remove excess moisture from the biomass without depending on factors such as light intensity or climate. However, this type of equipment requires vacuum pumps that drag the excess water out of the biomass to achieve maximum efficiency. This has shown promising results in removing large amounts of moisture in relatively short times (less than 5 h). The latter can lead to excessive energy use, making it unfavorable for the stability of the different molecules, especially phycobiliproteins. To date, the usage of a food-grade dehydrator has only been reported by Barajas-Solano [3]. A dehydrator uses low temperatures (40 °C) and longer times (>12 h) to remove the excess moisture in the sample gently. In this case, the operation conditions allow us to maintain the concentration and purity of C-PC, APC, and PE from a thermotolerant strain of *Oscillatoria* sp.

Design experiments are a group of statistical techniques that allow a more robust analysis of the impact of one (or several) variables on a specific process [23]. In this case, the results obtained allow the fast identification of the most favorable conditions without the need to perform many experiments. In comparison, experiments under a “One-Factor-At-the-Time” approach (or OFAT) may require large amounts of experimental data (including replicates), which, if not analyzed correctly, may generate responses that overestimate a critical factor or fail to identify the range of action of a variable that has not been previously studied. In this present study, the statistical analysis found that time (h) and the drying method are the only significant variables in the design, meaning that temperature in the case of the extraction of C-PE is not as critical as one may initially think.

This research tested if the extraction of C-PE is affected by the application of a drying method. The results show that it is necessary to reduce the total moisture content in the biomass, since one of the levels on “factor C” was the usage of fresh, undried biomass, the data obtained from this specific method showed the lowest concentration and purity among all the experiments, with values below 0.01 g/L and 0.2 (purity index). The latter confirms that the excess moisture acts as a natural barrier, which reduces the permeability of the buffer, reducing the overall efficiency of the process. Therefore, in the case of using the biomass obtained from *Potamosiphon* sp. as a source of C-PE, it is mandatory to gently remove at least a part of the overall moisture within the biomass, which, in turn, will allow the buffer penetration through the multi-layer cell wall and will remove as much as possible of the C-PE.

The latter is interesting since most food-grade dehydrator works at 40 °C as the lowest temperature; this low temperature will not generate a significant impact on the concentration and quality of total proteins, and hence phycobiliproteins, since proteins can be altered in temperatures over 50 °C, therefore, this condition will not significantly reduce the overall content of C-PE. This result agrees with those reported by authors such as Rezvani et al. [12], Silva et al. [13], Demarco et al. [14], and Stramarkou et al. [15], who tested alternative solutions for the drying of cyanobacterial biomass, where temperatures below 50 °C can be achieved using non-conventional drying techniques. The significant difference between the literature and the results obtained in this research is the time needed for moisture reduction. The time required for technologies such as a freeze dryer or spray dryer to remove almost all moisture can be between 12–24 h [35,36,37,38]. On the other hand, a solar dryer or a cast-tape dryer can obtain the same result in less than six hours [12,13,14,15]. In comparison, the results of the surface response show that there is no statistical difference between using 12 or 24 h of drying. However, a new design can be adjusted to test less drying time (6–12 h), but the latter remains unclear until further experiments.

The other variable analyzed in this research is the purity index of the extracted C-PE. In this case, time (h) and the drying method also affect the purity of the extract. The purity index is a measurement to identify the presence of accessory proteins (pectin, globular proteins, and others) in the extracted sample. Typically, the concentration (in OD) of the selected phycobiliprotein is divided in the absorbance of the sample at 280 nm (C-PE = OD_562nm_/OD_280nm_). According to Patil [25] and Antello et al. [26], extracted phycocyanin with purity index values higher than 0.7 can be considered food grade. In this case, using a food dryer on the biomass *Potamosiphon* sp. allows obtaining extracts with values of up to 0.8, meaning that the crude extract can be considered food grade.

So far, most studies do not consider this type of data a critical variable since any available technique, such as salting-in/salting-out, can increase the purification of the extracted phycocyanin. However, this measurement is an exciting approach to understanding the behavior of other metabolites during the drying process, especially soluble molecules such as proteins that can reduce the initial quality of the extracted sample. In that case, the subsequent processes required to obtain a high-quality extract would need less energy and fewer chemicals, which would benefit the process’s global impact. Further research is necessary to identify if this variable can effectively reduce the time, energy, and materials required to purify this colorant.

## 5. Conclusions

This study indicates that a food-grade dehydrator can improve the drying of cyanobacterial biomass without diminishing the concentration of valuable compounds such as C-phycoerythrin (C-PE). The results showed that temperature is the main variable affecting the concentration and purity of C-PE while drying time is not critical. The proposed conditions allowed a highly concentrated extract with a purity index of 0.8, higher than the recommended for food-grade (0.7) extracts. Future studies should further analyze the best operation time and extractability of other metabolites, such as carbohydrates and lipids.

## Figures and Tables

**Table 1:** Variables evaluated with their levels for the drying of *Potamosiphon* sp.

Variables	Type	Coded Name	Low Level (−1)	Center (0)	High Level (+1)
Drying temperature (°C)	Numeric	A	40	55	70
Drying time (h)		B	12	18	24
Drying method	Categoric	C	Dehydrator	Oven	fresh

**Table 2:** Resolved design for the drying of *Potamosiphon* sp.

Block	Run	Drying Temperature (°C)	Drying Time (h)	Drying Method
Block 1	1	40	12	Dehydrator
	2	70	12	Oven
	3	70	24	Dehydrator
	4	70	24	Dehydrator
	5	55	12	Dehydrator
	6	70	12	Oven
	7	40	24	Oven
	8	40	24	Oven
Block 2	9	59.03	24	Fresh
	10	40	12	Oven
	11	59.03	24	Fresh
	12	55	24	Oven
	13	55	18	Dehydrator
Block 3	14	40	24	Fresh
	15	70	24	Oven
	16	55	18	Dehydrator
	17	70	17.88	Fresh
Block 4	18	40	18	Oven
	19	55	12	Fresh
	20	55	12	Fresh
	21	70	18	Dehydrator
	22	55	18	Oven
Block 5	23	55	12	Oven
	24	70	12	Dehydrator
	25	40	16.44	Fresh
	26	40	24	Dehydrator

**Table 3:** Analysis of variance (ANOVA) of the model obtained for C-PE extraction.

C-PE (g/L)		**Sum of Squares**		**Df**	**Mean Square**		**F-Value**	***p*** **-Value**
	Block	6.18		4	1.54			
	Model	30.02		4	7.51		225.98	<0.0001 *
	A-Temperature	0.2471		1	0.2471		7.44	0.0143 *
	B-Time	0.0112		1	0.0112		0.3378	0.5687 **
	C-Drying method	30.02		2	15.01		451.87	<0.0001 *
	Residual	0.5647		17	0.0332			
	Lack of Fit	0.2003		12	0.0167		0.2290	0.9830 **
	Pure Error	0.3644		5	0.0729			
	Cor Total	36.76		25				
		R²	Adj R²	Pred R²	Adq Pr	Std. Dev.	Mean	CV %
		0.9815	0.9772	0.9577	28.5427	0.1823	−3.24	5.62

* Significant. ** Not Significant.

**Table 4:** Analysis of variance (ANOVA) of the model obtained for C-PE purity.

C-PE purity (Purity index)		**Sum of Squares**		**Df**	**Mean Square**		**F-Value**	***p*** **-Value**
	Block	0.0475		4	0.0119			
	Model	0.3902		3	0.1301		24.11	<0.0001 *
	A-Temperature	0.0695		1	0.0695		12.88	0.0021 *
	C-Drying method	0.0971		18	0.0054			<0.0001 *
	Residual	0.0722		13	0.0056		1.12	
	Lack of Fit	0.0249		5	0.0050			0.4881 **
	Pure Error	0.5348		25				
	Cor Total	0.0475		4	0.0119			
		R²	Adj R²	Pred R²	Adq Pr	Std. Dev.	Mean	CV %
		0.8007	0.7675	0.5783	10.9723	0.0734	0.5787	12.69

* Significant. ** Not Significant.

**Table 5:** Best drying conditions for extraction and purity of C-PE.

Coded Name	Variable	Units	Value
A	Drying temperature	°C	40
B	Drying time	h	12
C	Drying method	--	dehydrator
Z_1_	C-PE	g/L	0.11
Z_2_		Purity Index	0.82

## Data Availability

Not applicable.
